# Polyamine metabolism genes of maize (*Zea mays*) downregulated during compatible interaction with *Meloidogyne arenaria*

**DOI:** 10.1007/s11033-025-11153-3

**Published:** 2025-10-16

**Authors:** Arnika Przybylska

**Affiliations:** Institute of Plant Protection – National Research Institute, Poznań, Poland

**Keywords:** Root-knot nematode, Spermine synthase, Spermidine synthase, Polyamine oxidase, S-adenosylmethionine metabolism

## Abstract

**Background:**

*Meloidogyne arenaria* is one of the most economically important root-knot nematode (RKN) species with a wide host range, including maize. Although analyses of host-nematode interactions have highlighted the involvement of polyamines (PAs) in plant defense, the roles of spermine and spermidine synthases, crucial enzymes in PAs’ biosynthesis, and polyamine oxidase, which is involved in PAs’ catabolism, have not yet been investigated in the maize-RKN pathosystem. Thus, this study aimed to analyze the expression levels of genes encoding these enzymes during the compatible interactions between *M. arenaria* and maize (*Zea mays* L.).

**Methods and results:**

Time course analysis was performed on maize root samples collected at three time points. Total RNA was extracted from roots and used as a template for cDNA synthesis. The relative expression level of spermine and spermidine synthases and polyamine oxidase encoding genes was analyzed using a real-time PCR assay and normalized to the reference genes (*Leunig* and *FPGS*). In the case of the spermine synthase coding gene, significant upregulation was first observed, followed by downregulation. On the other hand, in the spermidine synthase coding gene, significant downregulation in all analyzed time points was reported. Significant downregulation was also observed at 8 dpi in the polyamine oxidase coding gene.

**Conclusions:**

The obtained results suggest that the downregulation of genes encoding proteins involved in PAs’ biosynthesis and catabolism may partially contribute to the nematode-induced suppression of some host defense response mechanisms, which in turn may be involved in facilitating RKN colonization of the susceptible host.

## Introduction

From approximately 100 described root-knot nematodes (RKNs), *Meloidogyne arenaria*, along with *M. hapla*, *M. incognita*, and *M. javanica*, ranks among the most economically important species [1]. It exhibits a broad host range, infecting both monocotyledonous and dicotyledonous plants, including numerous crop species, with maize (*Zea mays* L.) being one of its primary monocot hosts [2]. During the invasion by RKNs, the process of transcriptional reprogramming is induced, leading to the formation of giant cells, which are essential for the nematodes’ feeding and development [3]. The symptoms of RKN infection primarily appear below ground and are characterized by the presence of galls on roots, tubers, and peanut pods [4].

During the plant–nematode interactions, many molecular changes are induced in the host, including alterations in its transcriptome and proteome [5]. This leads to changes in the expression of a range of genes important for host defense and associated with providing conditions necessary for the nematode’s transition to subsequent developmental stages and reproduction [6–8]. Additionally, changes in enzyme activity were reported, e.g., ribonucleases or peroxidases [9]. In the plant invasion by RKNs, the effectors are crucial for successful infection. Effectors are small proteins produced in the nematodes’ glands, primarily in their invasive stage J2, sometimes also in the larval stages J3/J4, and introduced along with secretions from the stylet directly into the plant tissue. Effectors can suppress plant response in various ways and may be specific to one nematode species as well as conserved among several different RKNs species [10].

In our previous study, we described the first potential effector protein for *M. arenaria*, MaMsp4, and indicated that its molecular partners from maize play a role in plant defense response and modifications of the plant cell wall [11]. We also discovered that one of the maize proteins, interacting with MaMsp4, is S-adenosylmethionine decarboxylase, a key enzyme in polyamines (PAs) - spermine and spermidine biosynthesis [11]. We also observed an increased abundance of S-adenosylmethionine synthase during the analysis of the maize proteome after *M. arenaria* infection [12].

The hypothesis of this study assumes that the expression levels of genes encoding proteins involved in PAs' metabolism are changing during compatible interactions between *M. arenaria* and its monocotyledonous host, maize (*Zea mays* L.). The role of PAs was described during other host–RKN interactions. During incompatible plant–RKN interactions, the upregulation of proteins related to PA biosynthesis was observed in *M. chitwoodi*-resistant *S. tuberosum* plants at four time points after infection [13]. Moreover, some other studies suggest that exogenously applied PAs may improve plant resistance to *Meloidogyne* infection [14, 15]. On the other hand, during compatible interactions, spermidine synthase in *Arabidopsis thaliana* was reported to be a target for cyst nematode, *Heterodera schachtii* effector protein – Hs10A06. The authors suggested that the secretion of Hs10A06 into the plant leads to an accumulation of spermidine, which in turn triggers an increase in polyamine oxidase (PAO) activity [16]. PAO catabolizes spermine and spermidine to produce 4-aminobutanal and N-(3-aminopropyl)−4-aminobutanal, respectively, as well as hydrogen peroxide (H_2_O_2_) [17].

This study aimed to analyze the expression levels of genes encoding spermine and spermidine synthases as well as polyamine oxidase 1 (ZmPAO1) in a maize variety susceptible to *M. arenaria* infection at three time points after inoculation: 24 h post-inoculation (hpi), 3 days post-inoculation (dpi), and 8 dpi. Time points were selected to correspond to the early stage of nematode infection, when the migratory J2 larval stage of *M. arenaria* can still be observed and before any visible symptoms on roots appear. According to the results published by Velloso et al. [18], J3 sedentary larvae of various *Meloidogyne* species begin to appear at 9 dpi in tomato plants. Moreover, in our previous studies, we observed J3/J4 larvae at 3 weeks after maize infection [11].

## Materials and methods

### Material

The materials for this study included one population of *M. arenaria*, kindly provided by the Flanders Research Institute for Agriculture, Fisheries and Food in Merelbeke, Belgium, and the PR39F58 maize variety (Pioneer), which is susceptible to *M. arenaria* infection. The nematode population was maintained on maize plants under greenhouse conditions at 26 °C. Their eggs were extracted from roots using the technique described by Hussey [19]. Second-stage juveniles (J2 larvae) were hatched from these eggs, and approximately 1000 specimens per plant were used for inoculation of 3–4 weeks old maize seedlings at the 4–5 leaves stage. Inoculated plants were grown in a greenhouse at constant day and night temperatures of 25 °C and 20 °C, under controlled light conditions, and at 30% relative humidity. The day/night temperature settings were applied at 7 a.m. and 7 p.m. Infection of the analyzed plants was confirmed when root symptoms appeared (after approximately 4–6 weeks).

### RNA extraction and cDNA synthesis

Total RNA was extracted from root samples consisting of three randomly selected fragments of adventitious roots per plant. Samples were collected at three time points: 24 hpi, 3 dpi, and 8 dpi in four biological replicates taken from infected plants, along with samples from healthy plants as controls. Two hundred nanograms of RNA from each sample, extracted from tissue using GeneMATRIX Universal RNA Purification Kit (EURx, Gdańsk, Poland), was taken for cDNA synthesis with a Maxima First Strand cDNA Synthesis Kit for RT-qPCR (Thermo Fisher Scientific, Waltham, USA) in a 20 µl final volume.

### Real-time PCR assay

RT-qPCR primers for amplification of *spermine synthase*, *spermidine synthase*, and *ZmPAO1* were designed in this study with sequences: ZmSpmnFw: GACAAAGGGAGGTGCGGATG, ZmSpmnRw: GGCCACATCGGGTTATTGAAG, ZmSpmdnFw: AAGGGTTCTGTCCGCTATGC, ZmSpmdnRw: TTAGCTTTTGTGGTGCCGTG, ZmPAO1Fw: GTACAGCGCAGACTACGTCA, and ZmPAO1Rw: TTCTCGAACTCCTGCCACAC. Target genes were selected from the NCBI (National Center for Biotechnology Information) database and sequences deposited in GenBank with accession numbers as follows: NM_001112372 for *spermine synthase 1*, NM_001155838 for *spermidine synthase 1*, and NM_001111636 for *polyamine oxidase 1* were chosen. The specificity of the designed primers to the target genes sequences was confirmed by BLAST analysis. Two previously evaluated reference genes for maize were used for normalization, *Leunig* and *FPGS* [20]. Real-time PCR comprised a 10 µl reaction mixture consisting of 5 µl of 2x iTaq Universal SYBR Green Supermix (Bio-Rad, Hercules, USA), 0.1 µM of each primer, 1 µl of cDNA template, and water. No template control samples were included as well. The reactions were carried out on a LightCycler 96 platform (Roche, Basel, Switzerland). Reactions were done in three technical and four biological replicates (*N* = 12). Normalization was performed using two reference genes, based on the geometric mean of the normalized values from each reference gene. Relative quantification was conducted employing the 2-ΔΔCq method in nematode-infected plants relative to the healthy controls. Statistical significance of results obtained for *M. arenaria-*infected plants compared to those for healthy plants was assessed individually for each time point using Student’s t-test for parametric data, with significance set at *P* < 0.05. Alternatively, the Mann-Whitney test was employed for non-parametric results. All analyzed data were calculated using GenEx 6.0 software (MultiD Analyses AB).

## Results and discussion

### Root-knot nematode infection leads to the downregulation of spermine and spermidine synthase-coding genes

**Fig. 1:**
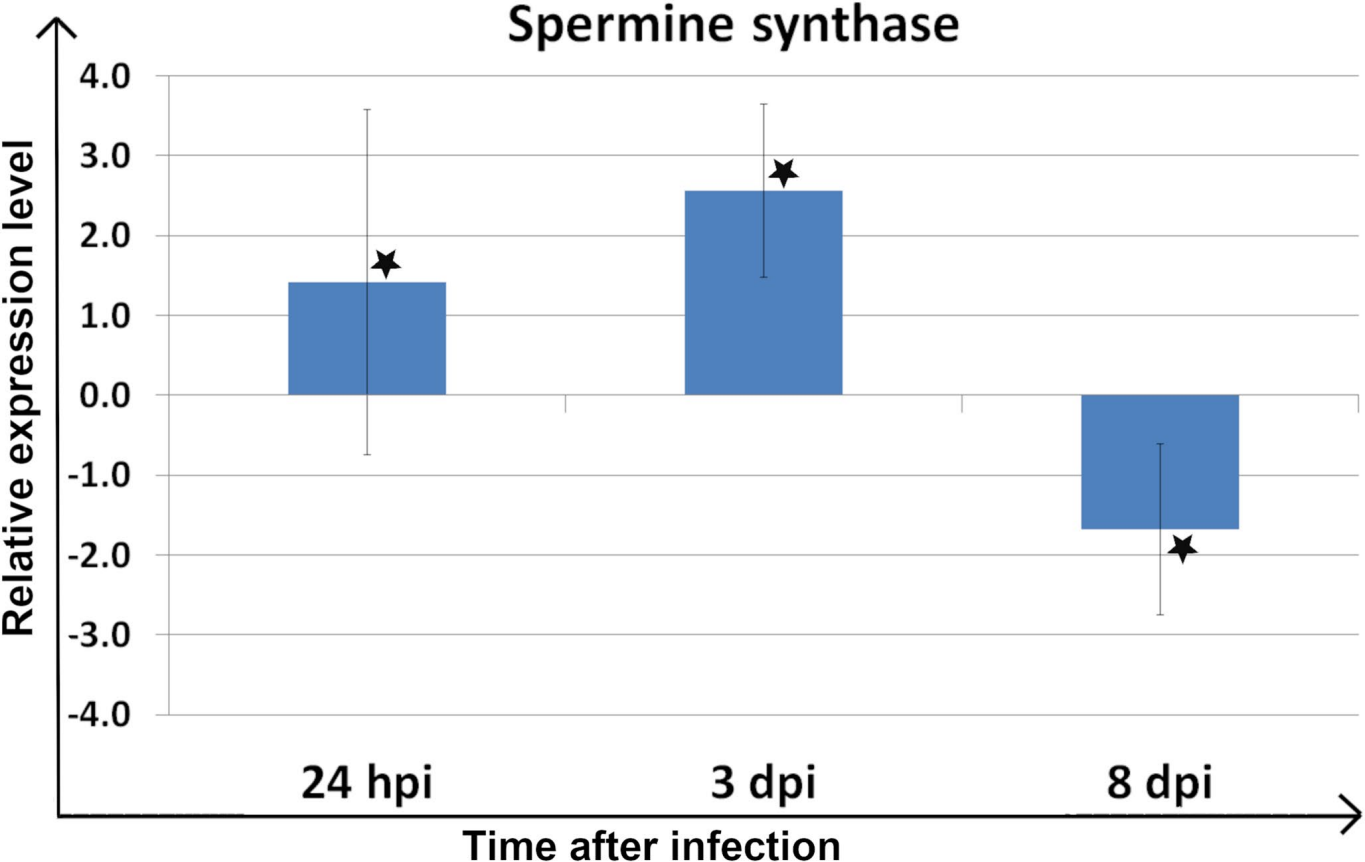
Relative expression level of the gene encoding spermine synthase across all biological and technical replicates. Asterisks indicate samples with statistically significant up- or down-regulation in *Meloidogyne arenaria-*infected plants compared to healthy plants (*P* < 0.05). Error bars represent a 95% confidence interval (CI)

As a result of the study, significant changes among samples collected at various time points post-inoculation were reported. In the spermine synthase coding gene, upregulation was observed at 24 hpi and 3 dpi, followed by downregulation at 8 dpi (Fig. 1; Table 1).

**Fig. 2:**
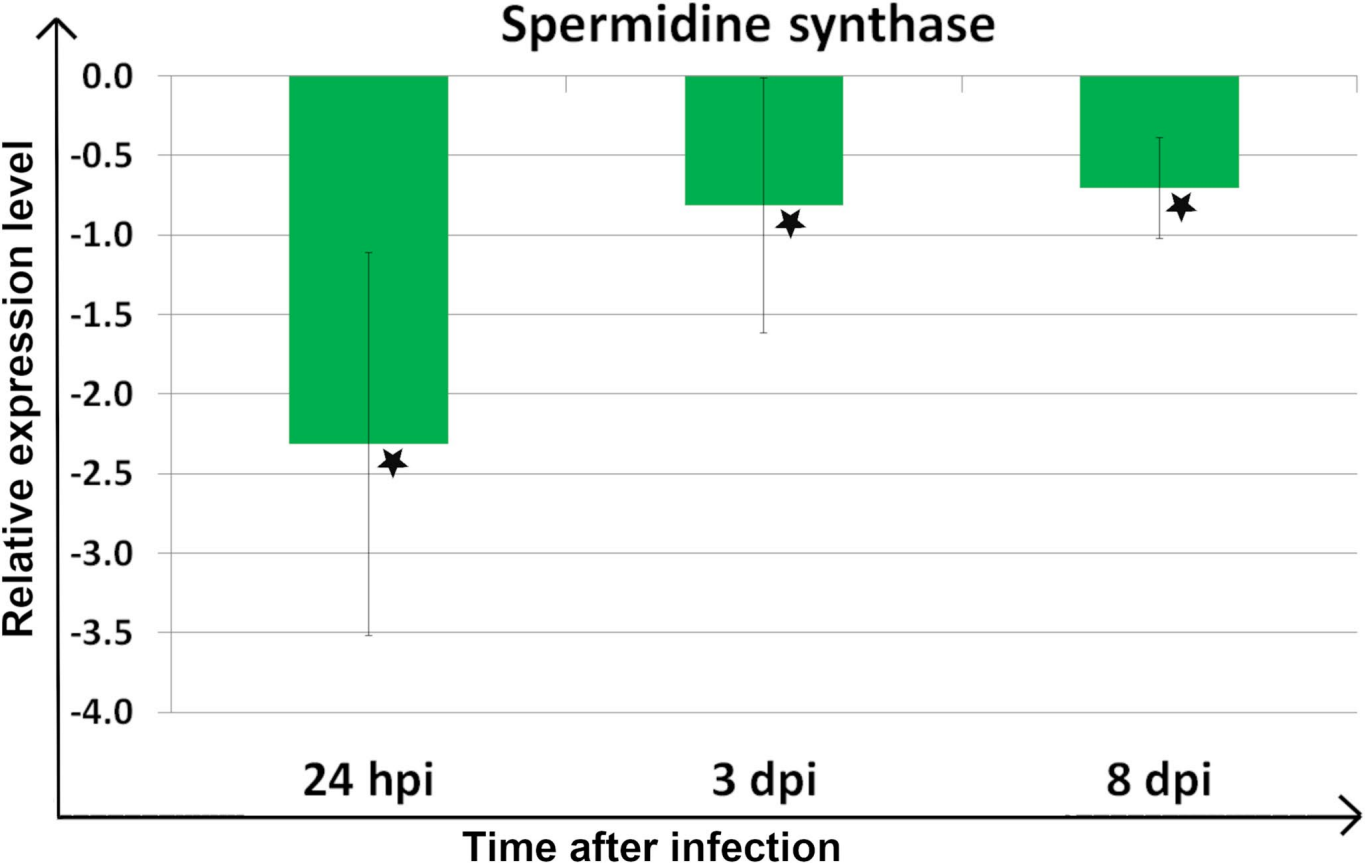
Relative expression level of the gene encoding spermidine synthase across all biological and technical replicates. Asterisks indicate samples with statistically significant up- or down-regulation in *Meloidogyne arenaria-*infected plants compared to healthy plants (*P* < 0.05). Error bars represent a 95% confidence interval (CI)

On the other hand, in the spermidine synthase coding gene, significant downregulation in all analyzed time points was reported, the strongest at 24 hpi (Fig. 2; Table 1).

### Root-knot nematode infection leads to the downregulation of the polyamine oxidase coding gene

**Fig. 3:**
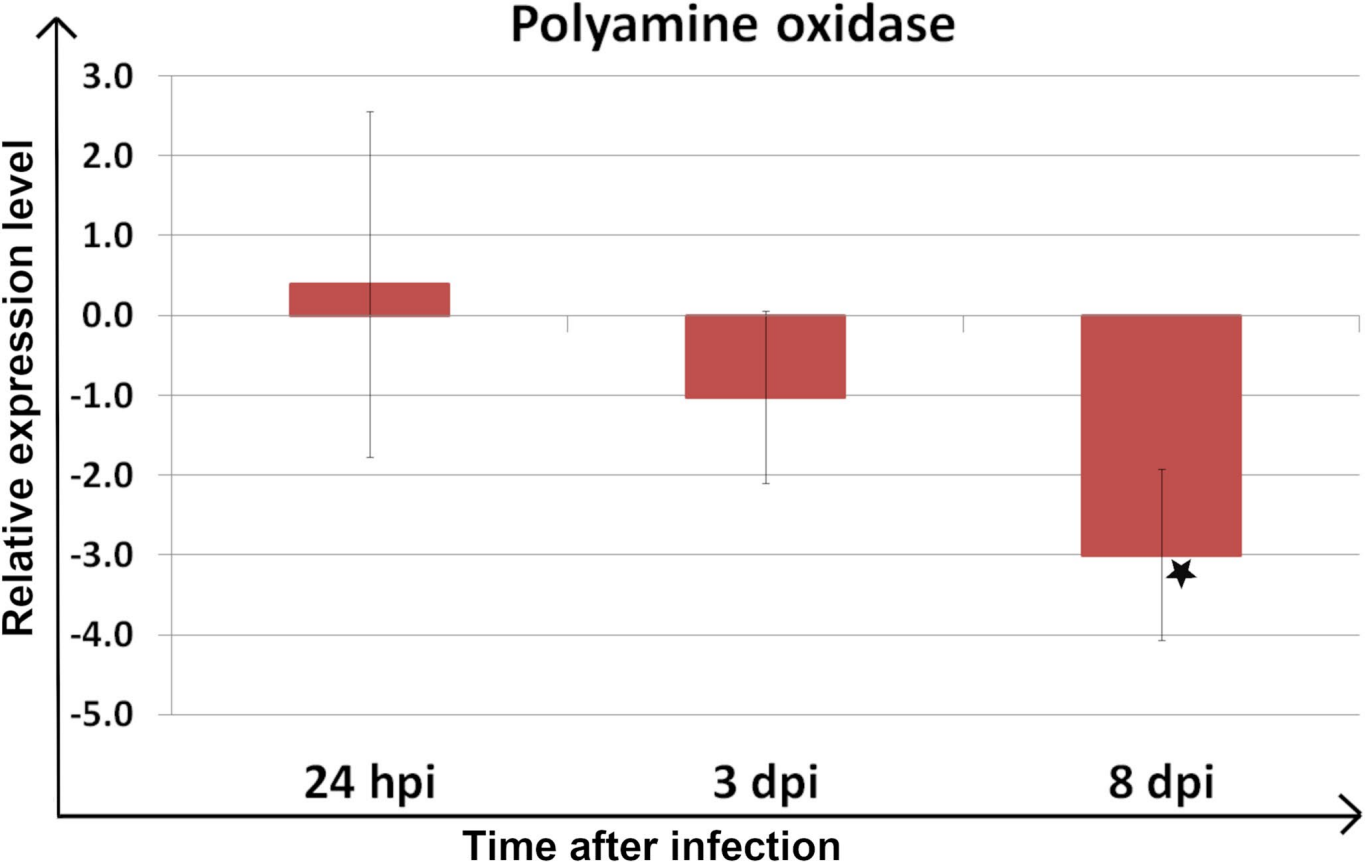
Relative expression level of the gene encoding polyamine oxidase 1 across all biological and technical replicates. Asterisks indicate samples with statistically significant up- or down-regulation in *Meloidogyne arenaria-*infected plants compared to healthy plants (*P* < 0.05). Error bars represent a 95% confidence interval (CI)

Statistically significant downregulation was also observed in ZmPAO1 coding gene expression level at 8 dpi (Fig. 3; Table 1).

**Table 1:** Log2 fold-change values for all three genes analyzed in the study. Asterisks indicate samples with statistically significant up- or down-regulation in *Meloidogyne arenaria-*infected plants compared to healthy plants (*P* < 0.05)

Gene	Log2 Fold Change		
	24 hpi	3 dpi	8 dpi
*Spermine synthase*	1,41*	2,56*	−1,68*
*Spermidine synthase*	−2,31*	−0,81*	−0,7*
*Polyamine oxidase*	0,39	−1,03	−3,01*

Plant PAs such as spermine and spermidine appear to play a crucial role in generating reactive oxygen species (ROS) in the catabolic reaction with PAO and facilitating their detoxification, thereby directly connecting them to the immune response [21]. Moreover, it has been observed that PAs accumulate during the activation of the plant’s resistance mechanism against various pathogens [22]. Spermine displays a unique role that distinguishes it from other PAs by orchestrating the induction and development of resistance reactions to diverse abiotic and biotic stresses [23]. Furthermore, Khajuria and Ohri [14] found that exogenously applied PAs help plants resist *M. incognita* infection by boosting stress tolerance, growth, and antioxidative defenses in tomato seedlings.

In this study, significant upregulation of the expression level of the gene encoding spermine synthase was observed, followed by downregulation during a susceptible variety of maize’s response to *M. arenaria* infection. Considering the current state of knowledge, the initial upregulation of spermine synthase during the early stage of infection can be interpreted as a plant defense response, which is subsequently suppressed by the nematode through its downregulation. In RKN-host interactions, expression of genes related to PA biosynthesis was described to be induced in the *M. chitwoodi-*resistant variety of *S. bulbocastanum* roots, which correlates with the nematode resistance response [13]. On the other hand, the gene encoding spermidine synthase was markedly downregulated at all time points, potentially due to suppression by certain nematode effectors, which frequently act as suppressors [24]. These data also partially correspond to the results of our previous study, in which the gene encoding S-adenosylmethionine decarboxylase, a key enzyme in spermine and spermidine biosynthesis, was downregulated in maize root and leaf samples during the early stage of *M. arenaria* infection, especially at 24 hpi, similarly to the spermidine synthase encoding gene from this study [11]. However, a higher abundance of S-adenosylmethionine synthase was observed in maize roots at 3 days post *M. arenaria* infection in our proteomic studies [12]. In contrast to compatible host-pathogen interactions, spermidine synthase has been reported to contribute to eggplant resistance against *Ralstonia solanacearum*, and the expression of its coding gene was upregulated during incompatible interactions. On the other hand, in the *H. schachtii*-*A. thaliana* pathosystem, where spermidine synthase is targeted by a nematode effector, spermidine synthase and S-adenosylmethionine decarboxylase encoding genes were upregulated, whereas the spermine synthase encoding gene was downregulated [14]. An increased expression level of the PAO-coding gene, as well as enhanced PAO activity, was also reported [14]. In this study, after initial upregulation of *ZmPAO1*, significant downregulation was observed. PAs’ catabolism contributes to ROS generation and scavenging, maintaining redox balance. Under stress, PAs’ catabolism increases ROS, especially H₂O₂, as a plant defense strategy [25]. Moreover, spermine was shown to modulate the balance between jasmonic acid (JA) and salicylic acid (SA) responses by stimulating JA biosynthesis and suppressing the SA pathway [26]. On the other hand, application of SA was reported to regulate PAs’ metabolism and influence ROS levels in plant tissue [27]. Considering the current state of knowledge on PAs’ catabolism, the observed downregulation of the ZmPAO1-coding gene may lead to suppression of ROS production and, in consequence, may contribute to the suppression of plant immunity. Interestingly, a few RKN effectors play a role as ROS suppressors, such as MgMO289 from *M. graminicola* [27].

The results described in this study are based on gene expression levels, which introduces certain limitations. To confirm the role of PAs’ metabolism in plant–RKN interactions, further functional analyses are required. Experiments such as measuring endogenous PA levels, quantifying ROS, or conducting assays on maize mutants would provide broader insight into the mechanisms underlying maize responses to *M. arenaria* infection.

## Conclusion

The results obtained in this study suggest that the downregulation of expression of *spermine synthase*, *spermidine synthase*, and consequently, *polyamine oxidase* during compatible interactions is crucial for the host to counter *M. arenaria* infection. The observed downregulation may be related to the suppression of ROS production and may partially contribute to nematode-induced suppression of host defense responses, which in turn facilitates RKN colonization of the susceptible host. Further analyses are necessary to confirm PAs’ role in ROS suppression in maize during RKN infection.

## Data Availability

No datasets were generated or analysed during the current study.

## Abbreviations

RKN: Root-Knot Nematode
J2: Juvenile 2
J3/J4: Juvenile 3/4
PAs: Polyamines
PAO: Polyamine oxidase
NCBI: National Center for Biotechnology Information
ROS: Reactive oxygen species
JA: Jasmonic acid
SA: Salicylic acid
